# Supplementary figures and images for: Massively parallel identification of functionally consequential noncoding genetic variants in undiagnosed rare disease patients
Source: Sci Rep. 2022 May 9;12:7576. doi: 10.1038/s41598-022-11589-8 (PMC9085742; doi:10.1038/s41598-022-11589-8)

Figure S1

**a**

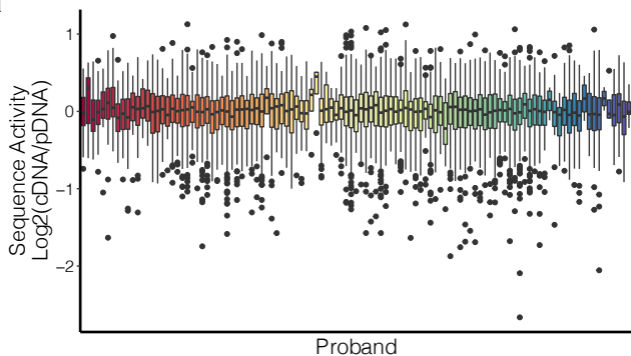

**b**

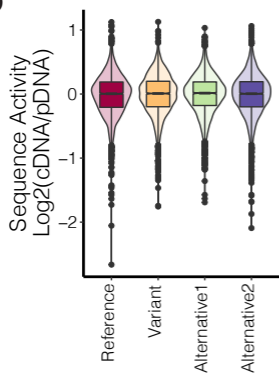

**c**

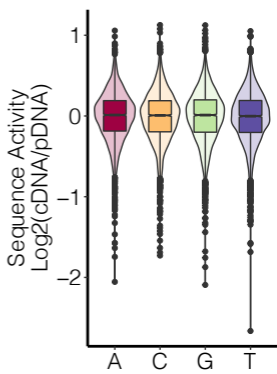

**d**

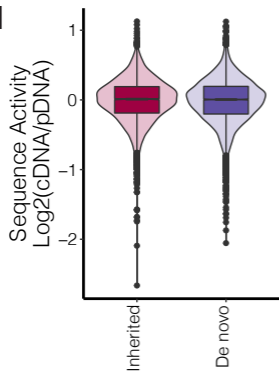

**e**

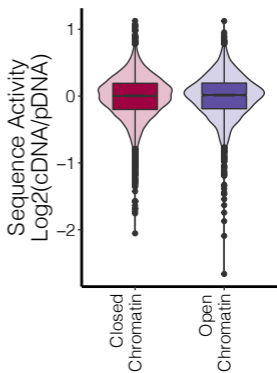

Supplement: Supplementary file 1 — Supplementary Figure S1. [file 41598_2022_11589_MOESM1_ESM.pdf]

Figure S2

**a**

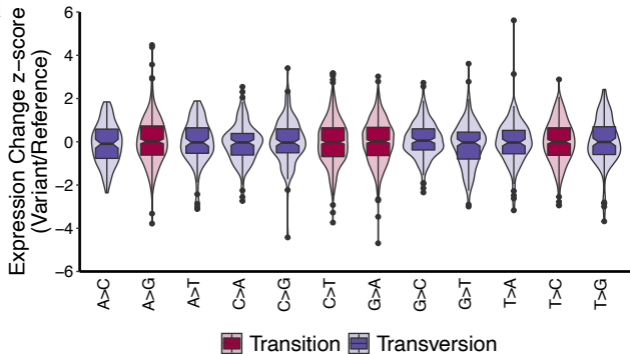

Supplement: Supplementary file 2 — Supplementary Figure S2. [file 41598_2022_11589_MOESM2_ESM.pdf]
