# Supplementary material for: Massively parallel identification of functionally consequential noncoding genetic variants in undiagnosed rare disease patients
Source: Sci Rep. 2022 May 9;12:7576. doi: 10.1038/s41598-022-11589-8 (PMC9085742; doi:10.1038/s41598-022-11589-8)
Supplement: Supplementary file 3 — Supplementary Legends. [file 41598_2022_11589_MOESM3_ESM.docx]

**Figure S1. MPRA sequence activity does not correlate with basic sequence features. a-e** Distribution of regulatory activity from genomic sequences profiled in MPRA separated by proband (**a**), allele type (**b**), base (**c**), inheritance type (**d**), and chromatin accessibility as determined by DNase I hypersensitivity (**e**).

**Figure S2. MPRA expression changes do not correlate with type of base mutation. a** Distribution of expression differences between reference and variant alleles profiled in MPRA separated by type of base mutation.

**Table S1. MPRA library design**

**Table S2. MPRA expression data**

**Table S3. MPRA fold change data**

**Table S4. Significance analysis of HPO term overlap**

**Table S5. RNA-seq fold change data**

**Table S6. PCR primers and conditions**
